# Specialty grand challenge: intensive training

**DOI:** 10.3389/fstro.2024.1412447

**Published:** 2024-04-26

**Authors:** Sandra A. Billinger, Richard Zorowitz

**Affiliations:** ^1^Department of Neurology, University of Kansas Medical Center, Kansas City, KS, United States; ^2^University of Kansas Alzheimer's Disease Research Center, Fairway, KS, United States; ^3^Department of Physical Medicine and Rehabilitation, University of Kansas Medical Center, Kansas City, KS, United States; ^4^Department of Cell Biology and Physiology, University of Kansas Medical Center, Kansas City, KS, United States; ^5^MedStar National Rehabilitation Network, Department of Rehabilitation Medicine, Georgetown University School of Medicine, Washington, DC, United States

**Keywords:** stroke, recovery, rehabilitation, intensity, exercise, walking, physical activity

## 1 Introduction

In 2017, there were over 7.6 million new ischemic strokes and 3.4 million new intracerebral hemorrhages globally. Stroke was the third-leading cause of death and disability combined (as expressed by DALYs [adjusted life-years lost]) in the world. The estimated global cost of stroke was approximately US$861 billion, or 1.12% of the global GDP (Feigin et al., 2022). Thus, because of the high burden of care associated with stroke, it is incumbent on rehabilitation providers to provide the best possible interventions to facilitate recovery in order to reduce the direct and indirect costs of post-stroke care.

When prescribing any stroke rehabilitation intervention, one of the key principles that contributes to neuroplastic changes in the brain is intensity (Kleim and Jones, 2008). Researchers have proposed variable definitions of intensity, including numbers of minutes, repetitions, sessions, or exercise intensity. While the American College of Sports Medicine uses a standard definition of exercise intensity (Table 1; Feito and Fountaine, 2022), the parameters used for moderate and vigorous intensity in stroke rehabilitation may vary across studies.

**Table 1:** Adapted from the american college of sports medicine's (ACSM) guidelines for exercise testing and prescription, 11^th^ edition (Feito and Fountaine, 2022).

**Intensity**	**Percentage of age-predicted maximal heart rate**	**Rating of perceived exertion (6–20)**
Very light	<57	<9
Light	57–63	9–11
Moderate	64–76	12–13
Vigorous (high)	77–95	14–17
Near-maximal to maximal	≥96	≥18

Aerobic exercise should be prescribed to stroke survivors as it may reduce modifiable vascular and metabolic risk factors of recurrent stroke, such as systolic blood pressure, fasting glucose, and LDL cholesterol (Billinger et al., 2014; Brouwer et al., 2021). The ACSM recommendations for healthy individuals are the same as those for stroke survivors: a minimum of 30 min of moderate-intensity exercise 5 days per week, or a minimum of 20 min of high-intensity exercise 3 days per week (Feito and Fountaine, 2022). To prescribe aerobic exercise, one can calculate the percentage of maximal heart rate (% HRmax) and rating of perceived exertion, or complete a submaximal exercise stress test using a treadmill or total body recumbent stepper to determine predicted peak oxygen consumption (VO_2_) (Mackay-Lyons et al., 2020). Many gaps in the literature exist to guide clinical practice for employing the FITT (frequency, intensity, time, and type) principles in stroke survivors across the continuum of recovery. A useful publication, “Aerobic Exercise Recommendations to Optimize Best Practices in Care after Stroke” is a user-friendly set of recommendations to guide screening and prescription and was developed by stroke rehabilitation professionals (Mackay-Lyons et al., 2020).

## 2 High-intensity training

To improve walking for individuals with chronic stroke (>6 months post-stroke), high-intensity gait training has been recommended as best practice (Hornby et al., 2020). High-intensity gait training is defined as variable context stepping activities, such as walking overground, treadmill walking, walking on uneven/compliant surfaces, and stair climbing, with a cardiovascular response of 75%−85% of age-predicted maximal heart rate (Henderson et al., 2022). High-intensity gait training can be performed either continuously or through intervals. High-intensity interval exercise focuses on repetitive switching between high-intensity bouts and active or passive recovery bouts (Boyne et al., 2013). Superiority between high-intensity continuous and high-intensity interval training has not yet been established in the stroke population. However, high-intensity interval gait training has been found to result in greater improvements in walking endurance and speed compared to that from moderate-intensity continuous gait training (Boyne et al., 2023), as well as improve blood pressure (Batacan et al., 2016; Costa et al., 2018), vascular function (Ramos et al., 2015), and aerobic fitness (Foster et al., 2015; Hwang et al., 2016). The benefits of high-intensity gait training for subacute stroke survivors (1 week to <6 months post-stroke) are less established compared to that of chronic stroke survivors, but multiple studies have demonstrated its efficacy (Holleran et al., 2014; Hornby et al., 2016; Henderson et al., 2022). For stroke survivors with and without significant cardiovascular co-morbidities, high-intensity gait training should be considered as the primary intervention if improving walking is a functional goal. The incidence of serious adverse events in both subacute and chronic stroke survivors have been limited with high-intensity gait training as compared to that of usual care (Boyne et al., 2013; Hornby et al., 2015; Henderson et al., 2022). To advance the field of stroke rehabilitation and recovery, we challenge researchers to develop, validate, and implement evidence-based interventions that maximize the functional recovery of the stroke survivors we serve (Scheets et al., 2021).

## 3 Opportunities

Current opportunities in the rehabilitation and recovery research field are to:

- Establish a standard definition of high-intensity aerobic exercise for stroke survivors.
- Determine the optimal timing to implement high-intensity aerobic exercise in stroke survivors: acute, subacute, chronic stroke?
- Determine if and how different modes of high-intensity exercise affect stroke survivors.
- Establish optimal exercise prescription parameters using FITT principles.
  ∘ Likely variable for each individual and may need to consider precision medicine.

## Author contributions

SB: Conceptualization, Project administration, Writing – original draft, Writing – review & editing. RZ: Conceptualization, Writing – original draft, Writing – review & editing.
